# Human embryonic stem cells hemangioblast express HLA-antigens

**DOI:** 10.1186/1479-5876-7-27

**Published:** 2009-04-22

**Authors:** Grzegorz Wladyslaw Basak, Satoshi Yasukawa, Andre Alfaro, Samantha Halligan, Anand S Srivastava, Wei-Ping Min, Boris Minev, Ewa Carrier

**Affiliations:** 1 Rebecca and John Moore's Cancer Center, University of California, San Diego, La Jolla, CA 92093, USA; 2 Department of Hematology, Oncology and Internal Diseases, The Medical University of Warsaw, Warsaw, 02-097, Poland; 3 Salk Institute, Department of Stem Cells, La Jolla, CA 92093, USA; 4 Departments of Surgery, Microbiology/Immunology, Pathology, University of Western Ontario, London, Ontario, N6A 5A5, Canada

## Abstract

**Background:**

It has been suggested that the initial differentiation of endothelial and hematopoietic cells during embryogenesis occurs from a common progenitor, called hemangioblast (hB). We hypothesized that these cells with dual hematopoietic/endothelial potential could be used in future regenerative medicine.

**Methods:**

We used the two-step differentiation technology to generate bipotential blast cells from human embryonic stem cells (hES). This involved short differentiation in our *in vitro *EB system followed by differentiation in semisolid culture medium supplemented with mixture of cytokines.

**Results:**

The occurrence of blast-colony-forming cells (BL-CFC) during EB differentiation (day 0–6) was transient and peaked on day 3. The emergence of this event was associated with expression of mesoderm gene T, and inversely correlated with expression of endoderm gene FoxA2. Similarly, the highest BL-CFC number was associated with increase in expression of early hematopoietic/endothelial genes: CD34, CD31 and KDR. The derived colonies were composed of 30–50 blast cells on day 6 in culture. These cells had homogenous appearance in Wright-Giemsa stain, but to a different extent expressed markers of immature hematopoietic and endothelial cells (CD31, CD34, VE-cadherin, Flt-1) and mature differentiated cells (CD45, CD33, CD146). We found that some of them expressed fetal and embryonic globin genes. Interestingly, these cells expressed also HLA class I molecules, however at very low levels compared to endothelial and hematopoietic cells. The blast cells could be successfully differentiated to hematopoietic cells in a CFU assay. In these conditions, blast cells formed CFU-M colonies (63.4 ± 0.8%) containing macrophages, BFU-E colonies (19.5 ± 3.5%) containing nucleated red blood cells, and CFU-EM colonies (17.1 ± 2.7%) composed of macrophages and nucleated erythrocytes. Cells of CFU-EM and BFU-E colonies expressed both ε – and γ- globin genes, but not adult-type γ-globin. When in endothelial cell culture conditions, blast cells differentiated to endothelial cells which had the ability to take up Dil-Ac-LDL and to form complex vascular networks in Matrigel.

**Conclusion:**

1) Hematoendothelial precursors exist transiently in early embryonic development and form single cell-derived colonies; 2) their differentiation can be tracked by the use of chosen molecular markers; 3) blast colonies consist of cells having properties of endothelial and hematopoietic precursors, however the issue of their ability to maintain dual properties over time needs to be further explored; 4) blast cells can potentially be used in regenerative medicine due to their low expression of HLA molecules.

## Introduction

The first hematopoietic and vascular cells develop from extra-embryonic mesoderm in the murine yolk sac at day 7.5 of gestation [1,2]. Once formed, these early progenitors organize into blood islands that consist of primitive erythroblasts surrounded by a layer of endothelial cells [3]. Close association of these two lineages led us to the hypothesis that they must arise from a common endothelio-hematopoietic precursor called hemangioblast [4-6]. During embryonic life, next waves of hematopoiesis occur in the aorta-gonad-mesonephros region (AGM), fetal liver, and finally in the bone marrow. However, the possibility of primitive hematopoiesis in other embryonic sites has been suspected for a long time. Sequeira Lopez et al. demonstrated that multiple regions within the embryo are capable of forming blood before and during organogenesis [7]. Therefore, there seems to be a widespread occurrence of hemo-vasculogenesis, the formation of blood vessels accompanied by the simultaneous generation of red blood cells [1,7-9]. When a vascular lumen forms, the erythroblasts "bud" from endothelial cells into the forming vessel [7,8]. Understanding the intrinsic ability of tissues to manufacture their own blood cells and vessels has the potential to advance the field of organogenesis, regeneration medicine and tissue engineering [10].

Subsequently, several investigators have identified human embryonic stem (hES) cell-derived populations that display both hematopoietic and endothelial potential [11-14]. Hemangioblast was identified as the cell which gave rise to colonies of blast-like cells (BLCs) [12]. These BLCs expressed KDR and represented a transient population that preceded development of primitive erythroid lineage. Similarly, progenitor comparable to the BLCs has been identified in the early gastrulating mouse embryo [15]. Mapping studies revealed that the embryo hemangioblasts exist in highest numbers in the posterior region of the primitive streak. This observation further supported the notion that hematopoietic commitment is initiated prior to the formation of yolk sac and blood islands.

It is well known how the immune system responds to conventional cell, tissue and organ transplants. However, the immune response to ES cell-derived grafts is difficult to predict due to the lack of donor-type vasculature, endothelial cells and professional antigen-presenting cells (APCs) in cellular transplants. The specific rejection of transplanted organs and tissues is primarily mediated by T cells and occurs mostly because of allelic differences between graft and recipient at their polymorphic major histocompatibility complex (MHC) molecules called human leukocyte antigen (HLA) in humans. Two types of MHC molecules exist, class I and II, and their function is to present antigenic peptides to CD8+ and CD4+ T cells, respectively. While the MHC class II antigens are normally present only on macrophages, dendritic cells, B cells and thymic epithelial cells, the MHC class I molecules are constitutively expressed at various levels on the surface of all adult nucleated cells [16]. Up to 1% of peripheral T cells in each individual can cross-react with allogeneic MHC antigens on transplanted cells [17], and that is why T cell-mediated allorejection is a rapid and vigorous process, which is mostly supported by preexisting memory T cells that have less stringent requirements for activation. Data on immunological properties of human and murine ES cells and their differentiated derivatives are controversial, ranging from those claiming unique immune-privileged properties for ES cells to those, which contradict these conclusions. This indicates that much more research is required to definitively understand the immunological features of ES cell derived progenitors. In this study, we examined the expression profile of HLA molecules on the surface of human ES cells, EB cells and blast-like cells. We demonstrated extremely low levels of HLA-A2 expression in the undifferentiated H9 human ES cell line, somewhat elevated HLA expression on the EB cells, and a moderately elevated HLA expression on the surface of combined blast colonies cells, as well as on cells derived from individual blast colonies. Therefore, this study represents an important attempt to define the HLA antigen expression and the graft rejection issue of human ES cells and their progenitors at different levels of differentiation.

In context of the increasing focus on regenerative medicine and the potential for development of stem cell based therapies for human diseases, the characterization and functional analysis of early mesodermal cell populations and their immediate progeny-hemangioblast-is of particular interest [18]. Therefore, we hypothesized that dual endothelio-hematopoietic progenitor can be obtained from hES cell-derived mesodermal progenitors early in the embryonal development. We expected that these blast cells would be able to form colonies of functional cells with dual hematopoietic/endothelial potential. Low expression of MHC class I molecules would allow their engraftment against histocompatibility barriers, and thus future clinical applications.

## Methods

### hES cell culture and differentiation

The hES cell line H9 (registered as WA09 by the US National Institutes of Health) was purchased from WiCell Research Institute (WI, USA). Cells have been cultured on the feeder layer of mouse embryonic fibroblasts (MEFs, Global Stem Cell Technologies, USA) in the culture medium consisting of DMEM-F12 with Knockout Serum Replacement (20%), L-Glutamine (0.8 mM), 2-Mercaptoethanol (119 μM), Non-Essential Amino Acid Solution (1%), and human recombinant bFGF (10 ng/ml) (all from Invitrogen, CA, USA) in standard cell culture condition (37°C, 5% CO2) and split mechanically every 3^rd ^day. When the hES culture reached 75% confluence, cells were used for differentiation studies in embryoid body (EB) system. The hES cells have been detached mechanically and small clumps of cells were resuspended in serum-free Stemline II Hematopoietic Stem Cell Expansion Medium (Sigma) containing BMP-4 and VEGF (50 ng/ml of each) (Invitrogen, CA, US). After 48 hours of incubation, half of the culture media was replaced with the Stemline II media containing BMP-4 and VEGF (both at 50 ng/ml), SCF, Tpo, and FLT3 ligand (all at 40 ng/ml) (Invitrogen, CA, US). When EB culture was performed for longer than 3 days, half of the medium was replaced every 48 hours with fresh medium containing BMP-4, VEGF, SCF, Tpo, and FLT3 ligand at concentrations described above. In the majority of experiments, EBs were collected after 72 hours of culture and dispersed to single cell suspension by incubation with Trypsin (0.05%) and EDTA (Invitrogen), and passing through 22 G needle and 40 μm cell strainer. Single cells were resuspended in Stemline II medium at a concentration of 2–5 × 10^6 ^cells/ml and further diluted in Methocult SF H4436 semisolid medium (Stemcell Technologies, Canada) at ratio of 1:30. The above culture medium was supplemented with BMP-4, VEGF, Tpo, and FLT3 ligand (all at 50 ng/ml) and cultured in Low Attachment Plate (Corning). The growth of blast colonies was observed after 3 days. For further studies, the BCs were hand-picked into Stemline II medium and dispersed mechanically to single cell suspension.

### Hematopoietic differentiation of blast cells

The blast cells were resuspended in Methocult SF H4436 media supplemented with 0.5% of EX-CYTE (Millipore) and plated onto untreated 12-well tissue culture plate (Becton Dickinson). After 15 days, the morphology of the colonies was assessed under inverted microscope Olympus with phase-contrast, the pictures were taken with Canon Digital Rebel XTi camera and the number of colonies of different type was subsequently counted. The single colony-forming units (CFUs) were hand-picked and assessed either by RT-PCR or Wright-Giemsa staining (Camco Quik Stain, Fischer, US).

### Endothelial differentiation of blast cells

For endothelial differentiation, blast cells have been resuspended in EGM-2 complete media (Cambrex) and incubated in fibronectin coated plates (Becton Dickinson) for 5 days. To prove that fibronectin-adhering cells are of endothelial lineage, the Dil-Ac-LDL uptake assay was performed. The cells were incubated with 10 ug/ml Dil-Ac-LDL (R&D System) for 4 h, dissociated with Trypsin-EDTA and spun onto glass slides. After fixation with 4% paraformaldehyde (Fischer) in PBS for 5 min., the cells were counterstained with Hoechst 33342 (Invitrogen) and visualized under fluorescent microscope. Next, the capillary formation assay was performed. Endothelial cells had been resuspended in EGM-2 complete media and added onto the surface of solidified Matrigel (BD Biosciences). After 24 h of culture, the capillary formation was visualized under the inverted Olympus microscope with phase contrast, and pictures were taken using Canon Digital Rebel XTi camera.

### RT-PCR

RNA was isolated using RNeasy Mini Kit (QIAGEN) and cDNA synthesis was performed with SuperScript^® ^First-Strand Synthesis System (Invitrogen) using the oligo(dT) method according to manufacturers' protocols. In samples from single-colonies, cDNA was prepared using CellsDirect cDNA Synthesis Kit (Invitrogen). To perform semi-quantitative analysis, 5 ug of RNA from each sample were used, the β-actin bands were used as internal loading control and a minimum number of cycles were performed to maintain the linearity of reaction. The sequences and annealing temperatures for primers resulted from extensive literature search and are listed in Table 1. PCR reaction was performed using Taq PCR Core Kit (QIAGEN) in DNA Thermal Cycler 480 (PERKIN ELMER CETUS) and the product was visualized in 2% agarose gel. (Table 1)

**Table 1:** The sequences of primers, product length and annealing temperatures used in RT-PCR reactions

Gene	Forward primer	Reverse primer	Size (bp)	Annealing temperature
β-Actin	TTTGAATGATGAGCCTTCGTCCCC	GGTCTCAAGTCAGTGTACAGGTAAGC	129	59
T	TGTCCCAGGTGGCTTACAGATGAA	GGTGTGCCAAAGTTGCCAATACAC	144	59
FOXA2	CCATTGCTGTTGTTGCAGGGAAGT	CACCGTGTCAAGATTGGGAATGCT	196	59
NeuroD	CCCATGGTGGGTTGTCATATATTCATGT	CCAGCATCACATCTCAAACAGCAC	196	59
KDR	CCTCTACTCCAGTAAACCTGATTGGG	TGTTCCCAGCATTTCACACTATGG	219	59
CD34	AAATCCTCTTCCTCTGAGGCTGGA	AAGAGGCAGCTGGTGATAAGGGTT	216	59
CD31	ATCATTTCTAGCGCATGGCCTGGT	ATTTGTGGAGGGCGAGGTCATAGA	159	59
SCL	AAGGGCACAGCATCTGTAGTCA	AAGTCTTCAGCAGAGGGTCACGTA	104	59
PTCH	CGCTGTCTTCCTTCTGAACC	ATCAGCACTCCCAGCAGAGT	282	60
GLI1	CTCTGAGACGCCATGTTCAA	ATCCGACAGAGGTGAGATGG	282	60
ε-globin	CACTAGCCTGTGGAGCAAGATGAA	AATCACCATCACGTTACCCAGGAG	304	59
γ-globin	CGCTTCTGGAACGTCTGAGGTTAT	CCAGGAGCTTGAAGTTCTCAGGAT	370	59
β-globin	TGTCCACTCCTGATGCTGTTATGG	AGCTTAGTGATACTTGTGGGCCAG	302	59

### Immunostaining

For FACS analysis, blast cells were isolated, washed and stained with appropriate monoclonal antibodies for 20 minutes at 4°C. The antibodies included: CD45-PerCp, CD34-FITC, CD31-PE (from Becton Dickinson), CD146-AF647, CD144(VE-cadherin)-PE, Flt-1-PE (from R&D Systems), CD33-PerCp (eBioscience). The cells were acquired using BD FACSCalibur (Becton Dickinson) and analyzed with FlowJo software (Tree Star).

### Immunofluorescence microscopy

Carefully cleaned coverslips were incubated in poly-L-lysine (Sigma) and dried for 24 hours. H9 cells, EB (day 3) cells and BC (day 6) cells were harvested, washed in PBS, and were allowed to settle on the coated coverslips for 30 min at 37°C. The cells were then fixed in 1% paraformaldehyde for 30 min, washed with PBS, and the coverslips were blocked with 1% BSA for 60 min. Staining for HLA-A2 was performed with the FITC-conjugated antibody BB7.2 (BD Pharmingen) together with DAPI (Promega) for 2 hours at room T°. The coverslips were then washed with PBS and mounted with ProLong Gold mounting medium (Invitrogen) on pre-cleaned microscope slides. The slides were then dried overnight at room T° in dark and observed under a Nikon fluorescent microscope.

## Results

### Tracking the development of hES cell-derived hemangioblast

Based on current literature, hemangioblast represents a transient cell stage during human development, and a number of genes have been identified as indispensable for hematopoiesis and/or blood vessel formation. We hypothesized that hemangioblast arises early during embryoid body formation and further undergoes differentiation to more mature hematopoietic and endothelial progenitors. We also hypothesized that the blast stage is clearly associated with the emergence of expression of hematopoietic and endothelial genes.

**Figure 1:**
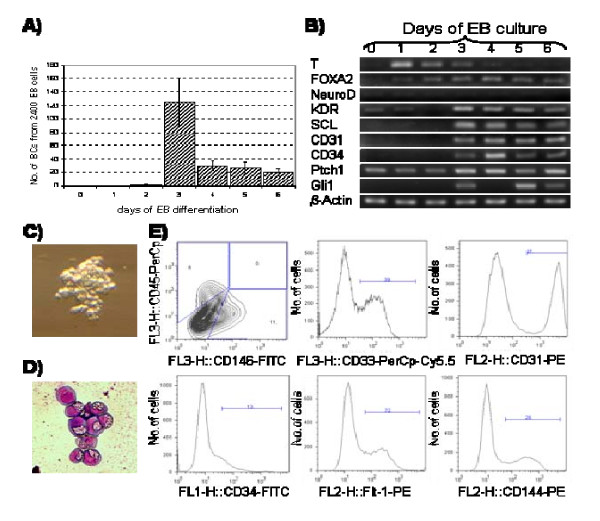
**Kinetics of hemangioblast formation in EB culture and characterization of blast cells**. A) Kinetics of blast colony (BCs) formation from cells derived from EBs on consecutive days of development. EBs were dispersed to a single-cell suspension and specific number of live cells was seeded in a semisolid medium. Colonies were counted on day 6 of BC culture. Experiment was performed in quadruplicates, and bars represent standard deviation (SD) from the mean. B) Dynamics of hemangioblast-related gene expression in EB differentiation system. Semi-quantitative RT-PCR was performed from RNA samples isolated from EBs picked on consecutive days of development. Input of RNA was normalized according to β-actin gene expression and minimal number of cycles was performed to achieve linearity of reaction. C) Blast colony on day 6 of culture (phase contrast, 100×). D) Blast cells on day 6 of blast culture (Wright-Giemsa stain, 200× light microscopy). E) FACS analysis of day 6 blast cells.

In order to find the exact time point when blast colony-forming cells (BL-CFCs) arise in the EB system, we started a series of BL-CFC cultures on days 0 to 6 of EB differentiation *in vitro*. In our hands, while only single blast colonies (BCs) were derived from day 2 EBs, there was a striking burst of BCs on day 3 followed by rapid decline in numbers (Figure 1A). On day 3, about 125 ± 35 out of 2400 EB cells formed BCs.

In order to define the correlation of hemangioblast formation with kinetics of gene expression, a semi-quantitative RT-PCR analysis was performed using RNA samples isolated from EBs at consecutive days of differentiation (Figure 1B). For analysis, we chose genes representing three germ layers (T-mesoderm, FOXA2- endoderm, NEURO D-ectoderm) and genes previously suggested to be closely related to hemangioblast (KDR, SCL, CD34, CD31). Moreover, we investigated expression of genes being a marker of hedgehog pathway activation (PTCH1, GLI1), as this pathway is implicated in early development of both hematopoiesis and vasculogenesis [19]. We observed that while T expression rapidly increased on day 1 of EB differentiation, it was gradually decreasing after day 1. On the other hand, the expression of FOXA2 was constantly increasing until day 4. In our culture conditions, we did not observe any significant expression of NEURO D; on day 3 of EB differentiation, we observed a significant increase in expression of KDR, SCL, CD34, CD31, PTCH1 and GLI1 genes. This was correlated with the appearance of highest number of BCs (Figure 1B).

BCs had a characteristic grape-like appearance and consisted of 30–50 loosely associated cells on day 6 (Figure 1C). These cells had homogenous morphology in Wright-Giemsa stain with big nucleus containing disorganized chromatin and narrow rim of cytoplasm filled with large-size granules (Figure 1D). However, as shown by FACS staining, they were quite heterogenous and to different extent expressed markers of both hematopoietic (CD34+, CD31+, CD45+) and endothelial cells (CD31+, CD34+, VE-cadherin+, Flt-1+, CD146+). At least a proportion of them were already committed to either endothelial (CD146+) or hematopoietic (CD45+) lineage (Figure 1E).

### Hematopoietic potential of blast cells

**Figure 2:**
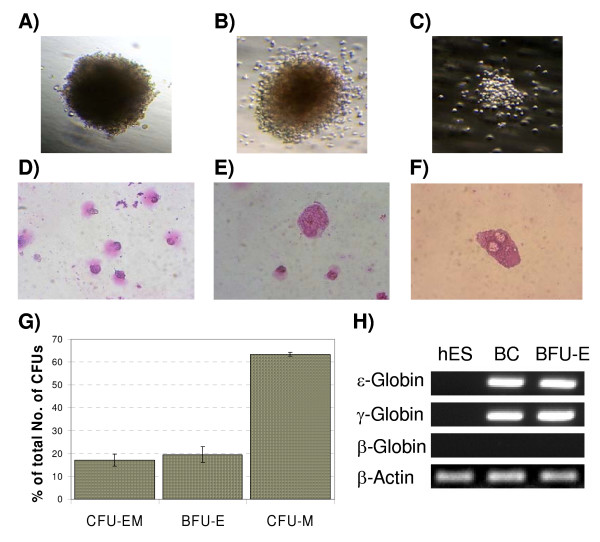
**Hematopoietic differentiation of blast cells**. Figures A-F show different types of hematopoietic colonies and cells derived from blast cells. A) burst forming unit-erythrocyte (BFU-E); B) colony forming unit- erythrocyte/macrophage (CFU-EM); C) colony forming unit-granulocyte/macrophage (CFU-GM) (40×, phase contrast); D) nucleated primitive erythrocytes from BFU-E; E) erythrocytes and macrophage derived from CFU-EM; F) macrophage derived from CFU-M (original pictures 200×). G) proportions of CFU colonies derived from blast cells. Bars represent standard deviations from the mean. H) analysis of globin genes expression in blast colony (BC), BFU-E and in undifferentiated hES cells (negative control).

The colony forming unit (CFU) assay is traditionally used to identify hematopoietic potential of certain cell populations. Characteristic morphology of derived colonies allows estimation of the type, number and differentiation stage of progenitor cells. Based on described phenotypes, we hypothesized that we can use CFU assay to characterize hematopoietic differentiation of EB-derived blast cells. In order to prove that, day 6 blast cells have been plated in Methocult H4436 medium. The morphology and number of colonies was estimated on day 15 after initiation of culture. In this assay, we obtained growth of three distinctive types of colonies (Figure 2A, B, C). The colony visualized on Figure 2A was solely composed of nucleated red blood cells and based on traditional nomenclature and colony appearance; it was called BFU-E. The colony shown in Figure 2B contained both nucleated erythrocytes and cells with macrophage morphology and was called CFU-EM. The third type of colonies was composed of macrophages only and therefore was called CFU-M (Figure 2C). Figure 2D, E, F represent nucleated pre-erythrocytes (Figure 2D, E) and macrophages (Figure 2F). The majority (63.4 ± 0.8%) of colonies were CFU-M, while BFU-E and CFU-EM colonies existed at similar proportions (adequately 19.5 ± 3.5% and 17.1 ± 2.7%) (Figure 2G). As we wanted to confirm if the observed erythropoiesis was of fetal or adult type, we performed RT-PCR analysis of globin genes from single colonies; both blast cells from single BCs and BFU-E colonies expressed only embryonic (ε) and fetal (γ) globin genes and not the adult-type β-globin (Figure 2H).

### Endothelial potential of blast cells

**Figure 3:**
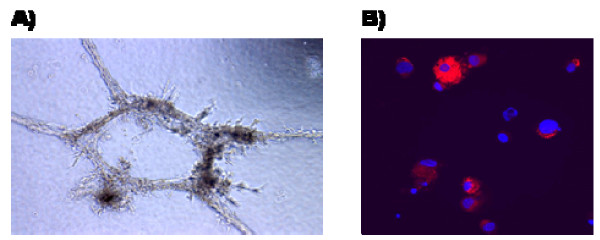
**Characterization of blast cell-derived endothelial cells**. A) vascular structures in Matrigel formed by endothelial cells after 24 h of culture (400×, phase contrast) B) Dil-Ac-LDL uptake by endothelial cells: red – Dil-Ac-LDL; blue- Hoechst (nuclei) (200×, immunofluorescence).

Based on the definition of hemangioblast, blast cells are the cells which can differentiate not only to hematopoietic progenitors, but also to functional endothelial cells, which are able to create vascular structures and pick up Dil-Ac-LDL. Therefore, we hypothesized that blast cells can be successfully differentiated to cells with properties of endothelium. In order to prove that, day 6 blast cells have been cultured for 4 days in endothelial cell medium on fibronectin-coated surface. The endothelial potential of differentiated cells which adhered to this surface was further assessed. After re-plating into Matrigel-containing wells, they spontaneously formed vascular-like structures after 24 hours of culture (Figure 3A). Moreover, they had the ability to take up Dil-Ac-LDL, which is a unique property of endothelial cells (Figure 3B). We concluded that blast cells have the ability to form endothelial progenitors as well as form vascular structures *in vitro*.

### HLA expression of hES cells, EB cells and blast-like colonies (BLCs)

**Figure 4:**
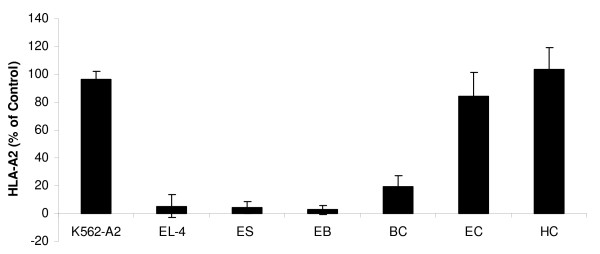
**Relative HLA-A2 expression**. Positive control cells K562-A2, negative control cells EL-4, undifferentiated ES cell line H9 (ES), EB cells (EB), blast colonies (BC), endothelial differentiated (EC) and hematopoietic differentiated (HC) cells were stained with the FITC-labeled anti-HLA-A2 antibody B B7.2 and relative immunefluorescence was quantified and expressed as a percentage of positive control.

To analyze expression of MHC-I proteins on the surface of human ES cells and their derivatives, we used monoclonal antibody BB7.2 directed against a subunit of the human leukocyte antigen-A2 (HLA-A2). Staining with this antibody revealed very low levels of HLA-A2 expression in the H9 human ES cell line. We also examined whether differentiation process of human ES cells would cause HLA-A2 upregulation. Differentiation of human ES cells into EBs resulted in a mild elevation of HLA-A2 protein expression (2- to 4-fold increase). Expression level of HLA-A2 proteins on the surface of combined blast colonies cells, as well as on cells derived from individual blast colonies was only moderately elevated. It is important to note, however, that the expression levels of HLA-A2 proteins on the surface of human ES-derived blast cells were still lower than those observed in the control human somatic cells. This lower level of HLA-A2 expression most likely reflects the relatively early nature of the blast cells derived from human ES cells (Figure 4), although they did explain potential to differentiate into endothelial and hematopoietic progenitors.

## Discussion

Future clinical applications of human ES cells and their progenitors will require that they do not express or express only low levels of HLA antigens, which can be tolerated by the host immune system. In this work, for the first time, we describe low expression of HLA antigens in human ES, EB, and blast cells with dual hematopoietic and endothelial potential, which may have future clinical applications.

Although some published data on the existence of murine and adult human hemangioblast exist [6], only recently two different research groups have used the hES/EB cell differentiation system *in vitro *to investigate human embryonic hemangioblast. Both Kennedy et al. [12] and Lu et al. [13] used ES/EB system to differentiate very early dual hematopoietic/endothelial precursors which were capable of formation of blast colonies (BCs). Although they applied different culture conditions and the phenotype of obtained blast cells significantly differed, in both cases, these cells could differentiate to both blood and endothelial progenitors.

Similar to the above publications, we performed hES cell differentiation in EB system and obtained blast colonies which were further shown to be bipotential. As the main scope of our studies was the evaluation of clinical application of blast cells, we adopted our culture conditions from Lu et al. [13] and studied HLA expression in these cultures. This methodology seems to be superior in order to not only investigate the existence of blast cells, but also to upscale its production. In the EB system, the early development of mesoderm and hemangioblast was stimulated with sequentially used growth factors: VEGF and BMP-4 in order to enhance mesodermal differentiation, and BMP-4, VEGF, Tpo, SCF and Flt3L to stimulate formation of early hematopoietic/endothelial precursors. We modified the ES-derived blast cell culture conditions using commercially available Methocult SF H4436 semisolid medium supplemented with BMP-4, VEGF, Tpo and Flt3L.

The blast colonies obtained by us had similar morphology as previously described, but they were composed of lower number of cells. Most likely this resulted from differences between hES cell lines used. Both Kennedy et al and Lu et al presented data based on H1 hES cells while we were using H9 cell line. As in the above papers, blast cells expressed embryonic and fetal globin genes, so at least some of them already differentiated to the erythroid lineage. Contrary to Lu et al., some of our ES-derived blast cells expressed CD31, CD34 and VE-cadherin, the molecules thought to be closely associated with the phenotype of hemangioblast [12]. However, some of the blast cells in culture were already terminally differentiated and were shown to express either exclusively hematopoietic marker CD45 or endothelial antigen CD146.

Despite this fact, the blast cells produced in our conditions could be successfully differentiated to either functional endothelial cells or blood cells. We observed growth of colony forming units composed of either primitive nucleated erythrocytes, macrophages or both these lineages. Therefore, our culture system most likely parallels very early yolk sac hematopoiesis where only these cell populations exist. The similar type CFUs were obtained by Kennedy et al. Contrary to Lu et al., we did not obtain growth of multilineage colonies containing also megakaryocytes and granulocytes, which may be due to the modification of culture conditions described in methods and materials.

In both reports, as well as in our studies, it was shown that the majority of colonies, but not necessarily single cells, are bipotential. This suggests that hemangioblast exists at the EB stage and gives rise to bipotential cell clone. But, are the single blast cells also bipotential? Lu et al. reported that cells from primary blast colonies can form secondary colonies and a proportion of them maintain bipotentiality. This means that at least some of the blast cells have properties of hemangioblast. We also investigated this issue, but the yield of secondary colonies was very low and the majority of them formed BFU-E colonies rather than blast colonies. Therefore, based on our observations, it is most likely that the majority of blast cells obtained at day 6 are already committed precursors of blood cells or endothelium. In this situation, the real hemangioblast seems to occur mainly at EB stage and is transient.

In order to prove how long cells persist in a hemangioblast or hemato-endothelial precursor stage, as well as how to optimize the yield of EB-derived blast cells, we performed an experiment with sequential formation of blast cells from EBs from day 0 to 6. Based on our data, it is clear that blast colony-forming cells (BL-CFCs) – or dual hemato-endothelial precursors arise early in EB development and are called hemangioblasts (day 3). Moreover, we performed semi-quantitative RT-PCR analysis of gene expression in developing EBs, confirming that the differentiation of BL-CFCs occurs just after differentiation of mesoderm layer and was suppressed by a subsequent development of endoderm. We also observed that the expression of a number of hemangioblast-related genes (CD34, CD31, KDR) peaks exactly at the time point when BL-CFCs aroused. Therefore they can be used in quantitative analysis of hemangioblast differentiation in EB culture (and in improved culture conditions) to obtain a higher yield of cells. The increased expression of genes of Hedgehog pathway signaling on day 3 suggests that their action may be related to the differentiation of early hemangioblasts. Based on the literature, Hedgehog signaling is important for embryonic hematopoiesis and vasculogenesis, and it was suggested that it enhances paracrine BMP-4 signaling, leading to the development of blast-like cells [19,20].

Blast cells differentiating from hemangioblasts or hemato-endothelial precursors appear at a very early stage of ES differentiation, and it is unclear from previous studies whether it expresses HLA molecules. In this work, we, for the first time, demonstrated that the blast cells express HLA molecules at an elevated level compared with their precursors: ES and EB cells. Other studies have also demonstrated low levels of expression of MHC class I molecules in human undifferentiated ES cells [21-24], while the levels of MHC class I molecules on human ES cells upon differentiation were reported to be slightly downregulated [21] or moderately upregulated [22]. These observations suggest that ES cell-derived therapeutics will most likely express MHC class I, and that they may be recognized by T cells and rejected upon transplantation. However, this issue still needs further detailed studies. Based on our data, although the blast cells can be characterized by mildly increased HLA expression compared to negative controls, e.g. ES and EB cells, it is still much lower than in differentiated endothelial and hematopoietic cells. Moreover, several published studies suggest immune- privileged properties of ES-derived cell products [23,25-28]. Human ES cells do not express co-stimulatory molecules and many other immune-related genes [24,29]. Moreover, the undifferentiated and differentiated ES cells were shown to be protected against T cell-mediated immune responses due to a high-level expression of the granzyme B inhibitor [28]. In addition, human and murine ES cells are capable of actively modulating immune reactions as demonstrated by their ability to inhibit third-party allogeneic dendritic cell-mediated T cell proliferation [23], to abrogate ongoing alloresponses in mixed lymphocyte reactions [26,30] and to completely prevent T cell cytotoxicity against allogeneic ConA blasts in vitro [31]. Although human ES cells express relatively low levels of MHC-I, it was shown that they were also insensitive to human natural killer (NK) cell-mediated cytotoxicity [22]. The resistance of hematopoietic stem cells to immune attack was shown in a previous study [32]. Notably, embryonic tissues from early gestational stages were also known to be less immunogenic than their adult counterparts [33]. In conclusion, we suggest that the ES cells and their early progenitors could evade immune surveillance due to their low immunostimulatory potential, and thus have future clinical potential.

## Conclusion

Based on current studies we conclude that hemangioblasts transiently exist at early ES/EB stage and then differentiate into blast cells. The bipotentiality of hemangioblast and blast cells provides the opportunity to use them in future cellular therapies of human disorders. Moreover, the blast cells can possibly find their application in the future regenerative medicine. They can successfully differentiate into endothelial cells and form vascular structures; therefore, they can potentially be used in different disorders where blood vessel structures are damaged physically or by inflammation, or when organs need rapid additional blood supply to maintain their functions (e.g. in case of heart infarction). For the first time, we have demonstrated low levels of HLA antigen expression in human blast cells, which supports their future clinical applications.

## Competing interests

The authors declare that they have no competing interests.

## Authors' contributions

EC contributed to conception and design, funding, supervision, data analysis and interpretation, final approval of the manuscript. GWB contributed to conception and design, collection and/or assembly of data, writing the manuscript. SY contributed to conception and design, collection and/or assembly of data. BM contributed to collection and/or assembly of data, writing the manuscript. AA contributed to collection and/or assembly of data. SH contributed to the drafting and critical revision of the manuscript. Wei-PM contributed to critical revision of manuscript, HLA studies. ASS contributed to conception and design of ES differentiation cultures.
